# Proposed Genetic Classification for the Skin Types: Helmy’s Skin Types Classification

**DOI:** 10.1097/GOX.0000000000002130

**Published:** 2019-04-23

**Authors:** Yaaser Helmy Ali

**Affiliations:** From the Faculty of Medicine, Al-Azhar University, Cairo, Egypt.

The author proposes a new classification for skin types, opposite to Fitzpatrick’s.^1^ This classification is based on the recent genome studies,^2^ which are proving that dark skin is the default human color. Human origins, according to anthropology researches,^3^ were found in the sunny east of the globe, followed by migration toward the less sunny west.^3^ Then genetic mutations^2^ have happened in the human genome of the west habitant, >4,000 years ago, to accommodate with the cloudy climate. This accommodation was described as genetic selection,^4^ when 3 genes were mutated for white skin and 1 gene has mutated for blue eyes.^5^ Two genes are *SLC24A5* and *SLC45A2* that lead to skin depigmentation and, therefore, Europeans’ pale skin today. The third gene, *HERC2/OCA2*, causes blue eyes, and it contributes to light skin color and the blonde hair.^2^

These mutations provided white populations the ability to synthetize vitamin D from little exposure to sun in cloudy climate and gave them the ability for milk digestion. In contrary to dark skin populations, who are living in very sunny latitude and hot weather, they are in need for large amount of melanin to protect their skin from ultraviolet damage.

As dark skin types are the earliest found on the Earth, it makes sense to have genetic classification for the skin types.

Although Fitzpatrick’s classification was described a long time ago and has clinical applications and therapy impact, this proposed classification is of genetic research importance. This proposed classification may make sense for genetic researches and prospective achievements during the management of genetic disorders in skin cancer and many others disorders of the skin. The proposed Helmy’s classification for genetic skin types is described in Table 1.

**Table 1.**
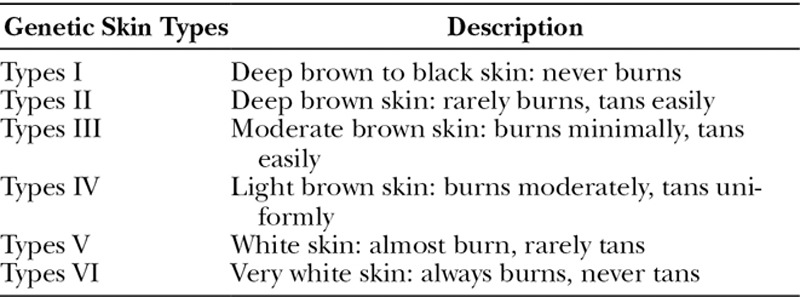
Proposed Genetic Skin Types
